# Effect of elevated embryonic incubation temperature on the temperature preference of juvenile lake (*Coregonus clupeaformis*) and round whitefish (*Prosopium cylindraceum*)

**DOI:** 10.1093/conphys/coad067

**Published:** 2023-08-30

**Authors:** Adam Harman, Hannah Mahoney, William Andrew Thompson, Meghan L M Fuzzen, Bhuvan Aggarwal, Lisa Laframboise, Douglas R Boreham, Richard G Manzon, Christopher M Somers, Joanna Y Wilson

**Affiliations:** Department of Biology, McMaster University, 1280 Main St. West, Hamilton, ON L8S 4K1, Canada; Department of Biology, McMaster University, 1280 Main St. West, Hamilton, ON L8S 4K1, Canada; Department of Biology, McMaster University, 1280 Main St. West, Hamilton, ON L8S 4K1, Canada; Department of Biology, McMaster University, 1280 Main St. West, Hamilton, ON L8S 4K1, Canada; Department of Biology, McMaster University, 1280 Main St. West, Hamilton, ON L8S 4K1, Canada; Department of Biology, McMaster University, 1280 Main St. West, Hamilton, ON L8S 4K1, Canada; Medical Sciences, Northern Ontario School of Medicine, Laurentian University, 935 Ramsey Lake Road, Sudbury, ON P3E 2C6, Canada; Department of Biology, University of Regina, 3737 Wascana Parkway, Regina, Saskatchewan S4S 0A2, Canada; Department of Biology, University of Regina, 3737 Wascana Parkway, Regina, Saskatchewan S4S 0A2, Canada; Department of Biology, McMaster University, 1280 Main St. West, Hamilton, ON L8S 4K1, Canada

**Keywords:** Behavioural thermoregulation, climate change, cold-water species, development, developmental plasticity, thermal effluent

## Abstract

Anthropogenic impacts can lead to increased temperatures in freshwater environments through thermal effluent and climate change. Thermal preference of aquatic organisms can be modulated by abiotic and biotic factors including environmental temperature. Whether increased temperature during embryogenesis can lead to long-term alterations in thermal preference has not been explicitly tested in native freshwater species. Lake (*Coregonus clupeaformis*) and round (*Prosopium cylindraceum*) whitefish were incubated at natural and elevated temperatures until hatching, following which, all groups were moved to common garden conditions (15°C) during the post-hatching stage. Temperature preference was determined at 8 months (Lake whitefish only) and 12 months of age (both species) using a shuttle box system. Round whitefish preferred a cooler temperature when incubated at 2 and 6°C compared with 0.5°C. Lake whitefish had similar temperature preferences regardless of age, weight and incubation temperature. These results reveal that temperature preference in freshwater fish can be programmed during early development, and that round whitefish may be more sensitive to incubation temperature. This study highlights the effects that small increases in temperature caused by anthropogenic impacts may have on cold-adapted freshwater fish.

## Teaser

Using lake and round whitefish, we tested whether incubation temperature altered the preferred temperature of fish later in life. Temperature preference was altered in round whitefish, fish reared in warmer temperatures preferred cooler temperatures at 12 months of age. Round whitefish were more sensitive to incubation temperature than lake whitefish.

## Introduction

Anthropogenic impacts have created scenarios where animals may be experiencing thermal stress during early critical life stages. Current predictions have proposed that the Great Lakes are expected to rise in temperature 4–6°C by 2100 (IPCC, 2014), at a rate of about 0.1°C/year (Austin and Colman, 2007). More immediate concerns arise from warmer effluent discharge from industrial practices that use natural bodies of water to remove waste heat. This effluent can lead to increases in local habitats by 1–3°C, particularly those along the shoreline (Thome *et al.*, 2016). Nearshore environments are critical regions for aquatic species, such as fish, providing regions to forage, shelter and breed (Hampton *et al.*, 2011). One risk for aquatic species that breed in these environments is the exposure of immobile embryos to supraoptimal temperature conditions throughout embryogenesis.

Environmental temperature exerts considerable control over the chemical process orchestrating development in fish (Stevens and Fry, 1970). Water temperature is a key determinant of growth in fish (Magnuson *et al.*, 1979; Jobling, 1981), with increased temperature during embryogenesis leading to accelerated growth and developmental rates (Gillooly and Dodson, 2000; Nytrø *et al.*, 2014; Sun and Chen, 2014). However, fish embryos display many plastic traits that can be influenced by their developmental environment (Jonsson and Jonsson, 2019). For instance, a positive relationship has been noted with incubation temperature and post-hatch metabolic rate in several fish species (Bozek *et al.*, 1990; Marty *et al.*, 1990; Barrionuevo and Burggren, 1999). Interestingly, the thermal optimum of key metabolic enzymes at the adult stage increases in response to temperatures experienced during rearing (Schnurr *et al.*, 2014). Taken together, this may be indicative of an increased need to elevate body temperature to meet changes in metabolic demands. As ectothermic poikilotherms, several studies have demonstrated that fish aggregate to their thermal preference (*T*_pref_; Kellogg and Gift, 1983; Reynolds and Casterlin, 1980; Stevens and Fry, 1970) to maintain their metabolic, growth and/or reproductive optimums (Larsson, 2005; Haesemeyer, 2020). *T*_pref_ has been shown to vary across life stage (Edsall, 1999), season (Mortensen *et al.*, 2007), time of day (Macnaughton *et al.*, 2018) and metabolic state (Killen, 2014). However, the impact of elevated incubation temperature and any long-term change to *T*_pref_, particularly in native cold-water fish, has not been explicitly tested.

Lake (*Coregonus clupeaformis*; LWF) and round (*Prosopium cylindraceum*; RWF) whitefish are cold-water-adapted species that have an extensive range across North America (Ebener *et al.*, 2010). These fish species serve an important ecological role in food webs and support commercial fisheries and indigenous communities (Ebener *et al.*, 2010). LWF and RWF occur sympatrically, co-existing due to differential habitat and resource usage within lakes (Eberts *et al.*, 2016). Both species of whitefish broadcast spawn in shallow (<10 m) cobble beds in late November, and embryos remain in these shallow waters until the ice melt in spring (April–May; Scott and Crossman, 1973). These long incubation periods coincide with temperatures of 0.5–2°C at these depths (Schwab *et al.*, 1999; Patrick *et al.*, 2013; Thome *et al.*, 2016), and these animals may be sensitive to increases in temperature imposed by anthropogenic impacts. Indeed, laboratory studies strongly support this, with whitefish exposed to elevated temperature during embryogenesis generally exhibiting perturbed morphology, precocious development and increased mortality (Price, 1940; Brooke, 1975; Patrick *et al.*, 2013; Eme *et al.*, 2015, 2018; Mueller *et al.*, 2015, 2017; Lee *et al.*, 2016; Lim *et al.*, 2017, 2018; Mitz *et al.*, 2019). These effects become more prevalent at constant temperatures ≥5° (Price, 1940; Brooke, 1975; Patrick *et al.*, 2013; Eme *et al.*, 2015; Mueller *et al.*, 2015; Lim *et al.*, 2017; Mueller *et al.*, 2017; Eme *et al.*, 2018; Lim *et al.*, 2018; Mitz *et al.*, 2019), with RWF appearing more sensitive, experiencing mortality rates 30–40% higher than LWF (Lim *et al.*, 2017; Lim *et al.*, 2018). Thermal stress during embryogenesis can augment and perturb the typical development of both LWF and RWF, but studies exploring impacts at post-hatch stages are limited. Work at later life stages is a necessity given that whitefish embryos could be exposed to temperatures as high as 5°C now and up to 8°C within ~30 years at the current rate of warming (Austin and Colman, 2007).

This study tested the hypothesis that elevated temperature during rearing could impact the resulting thermal preference of juvenile LWF and RWF. Elevated temperature can lead to lethality in embryos of these species, but sublethal effects, such as changes in length and weight (Price, 1940; Brooke, 1975; Lee *et al.*, 2016; Mitz *et al.*, 2019), may lead to altered performance and function at later life stages. To test this, we reared LWF at their optimum and natural rearing conditions (2°C) and elevated constant water temperatures of 5 and 8°C. As RWF are more sensitive to elevated incubation temperature and experience nearly 100% mortality at 8°C (Lim *et al.*, 2017; Lim *et al.*, 2018), we exposed RWF to 2 and 6°C, and a colder temperature (0.5°C), to see effects in a lower range of environmental temperatures. We assessed behavioural performance at 8 and 12 months for LWF, and 12 months for RWF, determining their *T*_pref_, velocity, total distance travelled and movement across temperature gradients. The results suggest that elevated incubation temperature can alter RWF *T*_pref_, but not LWF.

## Materials and Methods

### Study species

**Figure 1:**
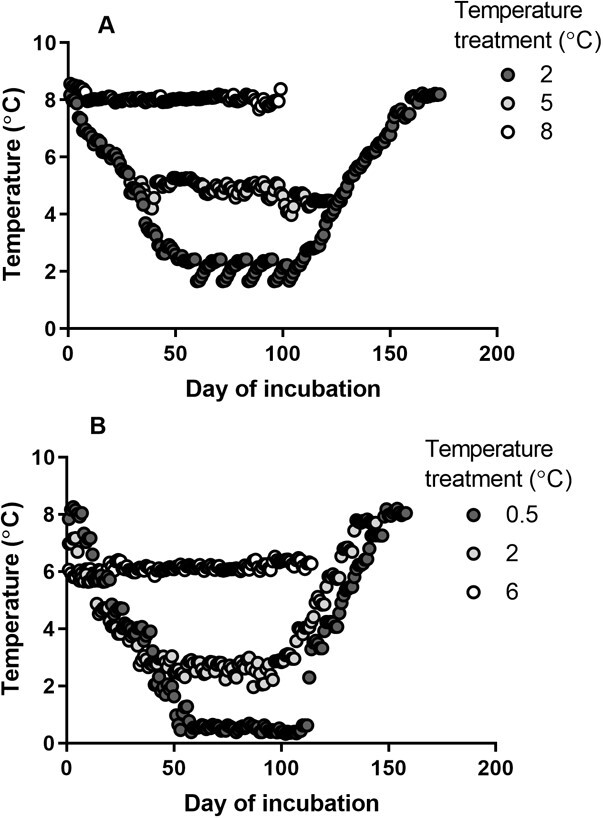
Daily temperature readings of incubation of lake whitefish (LWF; A) and round whitefish (RWF; B) until complete hatch of population. Data points represent averages of daily readings taken in 15-min intervals, with error bars excluded for visibility.

Fertilized LWF embryos were acquired from White Lake Fish Culture Station (Sharbot Lake, ON) on 30 November 2017 (reared to 12 months) or 27 November 2018 (reared to 8 months). Spawning RWF were obtained from Lake Ontario (Port Darlington, GPS 43°51′50″N 78°44′35″W) on 10 and 11 December 2018. RWF were stripped of eggs and milt and returned to the water. Artificial *in vitro* fertilization occurred immediately after stripping. Embryos were disinfected with Ovadine^®^ solution and transported in lake water back to McMaster University. Embryos (160–310) were plated into 200 mm × 20 mm sterile petri dishes containing 200 ml of dechlorinated city tap water, and then moved to incubators (Mitz *et al.*, 2014). Embryos were initially kept at 8°C and cooled (1°C/week; change in temperature occurred within 1 day) until they reached a base temperature of 2, 5 or 8°C for LWF or 2 or 6°C for RWF (Fig. 1). To create the treatment for the 0.5°C RWF embryos, we maintained an ice slurry within an incubator set to 2°C for 100 days. Incubation temperature was maintained for 100 days to replicate the winter period (December to March), following which, embryos were warmed (1°C/week) until they reached 8°C. To confirm temperature within each incubator, TidbiT^®^ temperature loggers were placed in 200 mm × 200 mm petri dishes with 200 ml of dechlorinated water. For the base temperature (excluding warming and cooling periods), LWF were exposed to 2.08 ± 0.3, 4.81± 0.3 and 8.05 ± 0.1°C, and RWF to 0.54 ± 0.2, 2.58 ± 0.2 and 6.13 ± 0.2°C. Median hatch for LWF occurred at 50 days post-fertilization (8°C), 108 days post-fertilization (5°C) and 158 days post-fertilization (2°C). Median hatch for RWF occurred at 88 days post-fertilization (6°C), 114 days post-fertilization (2°C) and 118 days post-fertilization (0.5°C). We calculated the cumulative growing degree days experienced by each group until median hatch using the formula described in Chezik *et al.* (2014), with a temperature of 0.5°C used as our base temperature (lowest temperature utilized in this study and assessed in Lim *et al.*, 2017 and Lim *et al.*, 2018). LWF experienced 567.22-, 519.43- and 379.04-degree days at median hatch when exposed to 2, 5 and 8°C, respectively. At median hatch, RWF experienced 258.75-, 350.57- and 498.75-degree days when exposed to 0.5, 2 and 6°C, respectively. Hatchlings (~10) were placed in 100 mm × 20 mm petri dishes with 100 ml of water at 8°C until successful exogenous feeding. Water in petri dishes was changed three times a week for embryos and daily for larvae. Larvae were transferred to 1- to 10-l recirculating tanks and warmed (1°C/week) to 15°C, where they remained until testing (8 or 12 months post-hatch). All treatment groups were maintained in common garden conditions once they were warmed to 15°C. Larval fish were initially fed *Artemia* nauplii and slowly transitioned to pellet feed [Otohime B1 (200–360 μm)–C2 (920–1410 μm) larval feed].

**Figure 2:**
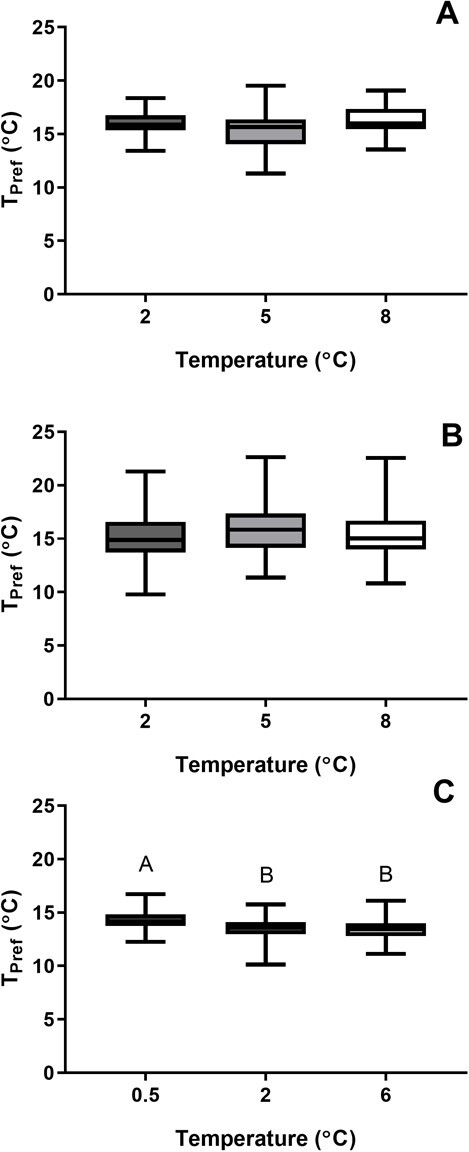
Ambient temperature during rearing can alter the thermal preference of fish in a species-specific manner. Boxplots comparing thermal preference (*T*_pref_) of (A) 8-month-old lake whitefish, (B) 12-month-old lake whitefish and (C) 12-month-old round whitefish after exposure to different ambient temperatures during embryogenesis. Lower and upper box boundaries are the 25th and 75th percentiles of the data, with the line inside the box representing the median, and error lines encompassing the entirety of the spread of data. Different letters above the boxplots denote significant differences between treatment groups.

### Behavioural assay—shuttle box

The shuttle box system (Loligo^®^), first described by Neill *et al.* (1972), consists of two cylindrical tanks connected by a small rectangular ‘shuttle’ to allow movement of animals between the tanks (see Supplementary Information Fig. S1). Each tank is designated as the increasing (INCR) or decreasing (DECR) side, indicating the direction of temperature change when fish occupy that tank. To regulate temperature, system water was pumped through heat-exchange coils in hot (28°C) and cold (4°C) water baths (60-l chest coolers) with mixing in separate buffer tanks for each side. A recirculator 1/4 horse power (HP) chiller, magnetic drive centrifugal pump (300/600/950 W at 0/10/20°C; VWR) and 2 × 400-W aquarium heaters were used to maintain the temperatures in the cold and warm bath, respectively. The shuttle box temperature probe can report temperature units to 0.01°C accuracy. Polystyrene insulation (1/2″) and foam insulation tape (1/4″) were used to prevent heat loss and maintain stable temperatures in the cold-water bath. System water flowed at 240 ml/min via gravitational pull through temperature probes and into the shuttle box where mixing between the two sides is minimized by counter-directional currents. The orientation of the INCR and DECR tanks and the side to which the fish would be introduced were randomized for each individual, using an online tool (random.org), to limit any potential bias introduced by visual cues or side preference. Whitefish of the appropriate treatment group were randomly selected from their home tank (15°C) and transported to the shuttle box system in blacked-out 1-l glass beakers to prevent undue stress. A plastic divider separated the two halves of the arena, which when removed, started the acclimation period. Fish (1 per trial) were acclimated to the arena in a static setting, with the two arenas maintained to 14 and 16°C with a hysteresis of 0.25°C. After 2 h in this condition, the fish were tracked using a USB 2.0 uEye Camera under infrared light (Loligo^®^ Infrared Light Tray), recording the position of the fish in the arena. The onset of warming or cooling occurred in response to whether the fish would be in the INCR or DECR tank, with the difference in temperature between these two sections being maintained at 2°C and warming or cooling (hysteresis = 0.1°C) occurring at a rate of 4°C/h. A maximum temperature of 23°C and a minimum temperature of 7°C were implemented to prevent exposure to extreme temperatures, which could cause stress or mortality (Edsall and Rottiers, 1976). *T*_pref_ was calculated by the software as the median occupied temperature; additional measurements calculated were velocity (cm/s), total distance travelled (cm), time spent in INCR/DECR, number of passages and avoidance temperature (temperature at which a passage between tanks occurred). Following the completion of the assay period, fish were removed and measured for total length and mass before returning to a separate home tank (15°C). Prior to experimentation, whitefish were fasted for 12–20 h to prevent fouling of the water and to standardize metabolic state. To account for any potential growth over the study duration, the order of sampling among treatment groups was randomized using an online tool (random.org). In total, 103 (12 months old) and 87 (8 months old) LWF and 83 (12 months old) RWF were tested for *T*_pref_ using the Loligo^®^ shuttle box system. Differences in treatment group sizes were due to differential mortality in holding tanks during rearing and were not due to experimentation. Prior to experimentation, power analyses were carried out to determine the optimal sample size within an acceptable power range (0.6–0.8; Harman *et al.*, 2021).

### Statistical analysis

Data are presented as mean ± SD unless otherwise stated. *T*_pref_, velocity, total distance travelled, time in arena, number of passages and avoidance temperature among groups were analysed using one-way analysis of variance (ANOVA), with Tukey’s honest significant difference (HSD) *post hoc* for comparisons among individual groups. To determine whether length or body weight influenced recorded *T*_pref_, a general linear model was performed. A comparison was performed to assess species-specific differences, with the 12-month *T*_pref_ of RWF and LWF compared using a two-tailed *t* test. Bonferroni correction was applied to correct for multiple comparisons of *T*_pref_, resulting in an *α* taken of 0.0125 for *T*_pref_ analyses with LWF (a total of four comparisons) and 0.025 for RWF comparisons (a total of two comparisons). We developed a relationship between time (s) and temperature change (°C) in the shuttle box to determine the upper threshold of the system. This was done to remove possible outliers, as certain individuals were too active for the shuttle box system to determine *T*_pref_ due to limitations in heating/cooling rates. Outliers were identified using the robust regression and outlier removal method (ROUT; Motulsky and Brown, 2006). The residuals of this fit were analysed for potential outliers and then subjected to ordinary least-squares regression after the removal of outliers. A total of four outliers were identified and removed using the ROUT method (2 × 8-month-old LWF, 2 × 12-month-old RWF). All statistical analyses were completed in R (version 4.0.0 “Arbor Day”), except for outlier identification, which was completed in GraphPad Prism (version 8.4.3). All data and R scripts used for analysis were uploaded to a public GitHub data repository (https://github.com/WilsonToxLab/Shuttlebox-Thermal-Preference).

## Results

There was not a significant effect of rearing temperature on *T*_pref_ on LWF of 8 months of age (Fig. 2A; *F*_[2,82]_ = 3.505; *P* = 0.0346). Upper and lower avoidance temperatures were similar among treatment groups (Table 1). However, we note a non-significant trend, with 8-month-old LWF in the 5°C treatment group displaying the lowest activity, travelling an average distance of 173 m, compared with just over 190 m at 2 and 8°C. There was no observable change in the number of transitions between arenas in the shuttle box in these fish (Table 1). Total body length was similar among all treatment groups, varying less than 1 mm on average (Table 1). Likewise, body weight was similar across treatment groups, with the largest difference (11%) between 2°C (1.13 ± 0.32 g) and 5°C (1.25 ± 0.39 g). Linear models were fit, including body weight and total length as fixed effects, to determine if there was a relationship between size and *T*_pref_. Model results (*P* = 0.068, *P* = 0.061) indicated there was no significant interaction between *T*_pref_ with total length or body weight.

**Table 1:** Behavioural output and whitefish characteristics of thermal preference experiment

Species (age)	Incubation treatment	*n*	Upper avoidance (°C)	Lower avoidance (°C)	Distance (m)	No. of passages	Length (mm)	Weight (g)
Lake whitefish (8 months)	2°C	31	17.54 ± 0.73	15.41 ± 0.81	193 ± 47	183 ± 113	55 ± 5	1.13 ± 0.32
	5°C	29	17.23 ± 1.09	14.93 ± 1.38	173 ± 67	146 ± 121	56 ± 6	1.25 ± 0.39
	8°C	25	17.74 ± 0.52	15.63 ± 0.57	191 ± 60	170 ± 124	55 ± 9	1.18 ± 0.51
								
Lake whitefish (12 months)	2°C	31	17.14 ± 2.45	14.01 ± 2.62	247 ± 112	51 ± 95	114 ± 12	10.59 ± 3.04
	5°C	40	17.72 ± 2.26	14.68 ± 1.94	196 ± 71	51 ± 65	114 ± 11	10.76 ± 3.59
	8°C	32	17.41 ± 1.85	14.24 ± 2.16	209 ± 97	31 ± 55	114 ± 8	10.28 ± 2.43
								
Round whitefish (12 months)	0.5°C	27	15.80 ± 0.75^a^	13.73 ± 0.75^a^	217 ± 44	212 ± 81	62 ± 5^a^	1.60 ± 0.45^a^
	2°C	31	15.29 ± 0.93^b^	13.18 ± 1.01^a,b^	189 ± 65	188 ± 105	55 ± 6^b^	*1.19 ± 0.35^b^
	6 °C	23	15.11 ± 0.90^b^	13.04 ± 0.79^b^	198 ± 41	204 ± 79	62 ± 5^a^	1.71 ± 0.52^a^

Fish were reared at different temperatures during embryogenesis, and then held in common garden conditions post-hatch. Lake whitefish and round whitefish embryos were brought in at 8°C and cooled at 1°C/week to the base temperature (incubation treatment); after 100 days of incubation, temperature was warmed at 1°C/week until they reached 8°C. This simulated natural conditions with different base incubation temperatures. After successful exogenous feeding, fish were warmed at 1°C/week to 15°C and were held at that temperature until experimentation in a shuttle box. Total sample size (*n*), avoidance temperatures (temperature when a passage between chambers occurs), distance travelled, total number of passages and length of weight of each experiment are shown. All values are mean ± SD, with different letters denoting significant differences among groups.

At 12 months of age, LWF from all treatment groups (2, 5 and 8°C) displayed similar *T*_pref_ (Fig. 2B; one-way ANOVA; *F*_[2100]_ = 0.0765; *P* = 0.468). Upper and lower avoidance temperatures were comparable among all treatment groups, suggesting 12-month-old LWF were avoiding temperatures below 14.3°C and above 17.4°C on average (Table 1). Average total length and body weight were similar across all treatment groups, varying less than 1 mm or 0.5 g, respectively. Linear models were fit, including body weight and total length as fixed effects, to determine if there was a relationship between size and *T*_pref_. Model results indicated that body weight (*P* = 0.0678) and total length (*P* = 0.0607) did not significantly affect *T*_pref_ at 12 months old (Table 1).

**Table 2:** Temperature preference (*T*_pref_) of juvenile lake whitefish of different ages

Age (years)	Age (months)	Size (g)	*T*_pref_ (°C)	Holding temperature (°C)	Source
0	4–5^a^	2.8	15.9	8-11	Edsall (1999)
0	5–6	1.1–1.7	17–18^b^	—^c^	Opuszynski (1974)
0	5–6	1.9	16.8	8–11	Edsall (1999)
0	8	1.13	16.04	15	Present study
1	12	10.59	15.27	15	Present study
1	12–13^a^	3.9	15.6	8–11	Edsall (1999)
1	12–13	5.7	10	—^c^	Opuszynski (1974)

Age is provided in years and months. Age in months was estimated for previously published studies by assuming median hatch occurs within March–April, as suggested by testing dates for 1-year-old fish. *T*_pref_ from the present study is reported from 2°C treatment groups only. Edsall (1999) used simulated lake water temperature during embryonic incubation. All other *T*_pref_ data were provided by Edsall (1999). Holding temperature refers to water temperature in home tanks from hatch until testing.

a ^a^Repeated measure on same cohort of fish.

b ^b^Temperature preference estimated via inspection by Opuszynski (1974).

c ^c^Information not available.

Temperature exposure during rearing affects the *T*_pref_ of 12-month juvenile RWF (Fig. 2C; one-way ANOVA, *F*_[2,78]_ = 5.509; *P* = 0.0058). The *T*_pref_ RWF juveniles incubated at 2°C (13.53 ± 1.14°C) and 6°C (13.39 ± 0.99°C) as embryos displayed significantly lower *T*_pref_ compared with those incubated at 0.5°C (14.27 ± 0.95°C; *P* = 0.0216 and *P* = 0.01, respectively), with no differences between the 2 and 6°C groups (*P* = 0.8764). This change in preference was reflected in recordings of upper and lower avoidance temperatures. Fish exposed to 0.5°C (15.8 ± 0.75°C) exhibited a higher upper avoidance temperature (one-way ANOVA; *F*_[2,78]_ = 3.51; *P* = 0.0347; Table 1), with both 2°C (15.29 ± 0.93°C) and 6°C (15.11 ± 0.9°C) exposed RWF significantly decreased in comparison (*P* = 0.0216 and *P* = 0.01, respectively). Lower avoidance temperatures exhibited a similar trend (one-way ANOVA; *F*_[2,78]_ = 3.676; *P* = 0.0298; Table 1), with 0.5°C (13.73 ± 0.75°C) treated RWF having lower avoidance temperatures than 2°C (13.18 ± 1.01°C) and 6°C (13.04 ± 0.79°C) treated fish, differing significantly when compared with 6°C fish (*P* = 0.03). Total distance travelled (one-way ANOVA, *F*_[2,78]_ = 1.885, *P* = 0.159) and number of passages (one-way ANOVA, *F*_[2,78]_ = 0.522, *P* = 0.596) were statistically similar among all treatment groups. Total length was not consistent among treatment groups (one-way ANOVA, *F*_[2,78]_ = 15.097, *P* < 0.0001) as juveniles in the 2°C group were significantly smaller than those in the 0.5 and 6°C treatment groups (*P* < 0.0001). However, total length was not significantly different between 0.5 and 6°C treatments (*P* = 0.643). Body weight followed the same trend as total length (one-way ANOVA, *F*_[2,78]_ = 11.374, *P* = 0.000045, Table 1), with the 2°C group significantly smaller in body weight on average than the 0.5 and 6°C groups (*P* = 0.0017 and 0.0001, respectively).

**Figure 3:**
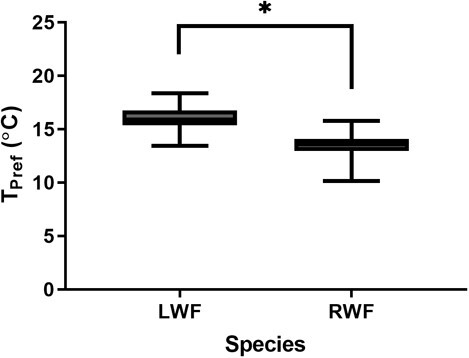
Species exhibit unique thermal preferences following rearing at a common temperature. Boxplots comparing thermal preference (*T*_pref_) of 12-month-old lake whitefish (LWF) and 12-month-old round whitefish (RWF) after experiencing 2°C during embryogenesis. Lower and upper box boundaries are the 25th and 75th percentiles of the data, with the line inside the box representing the median, and error lines encompassing the entirety of the spread of data. The symbol ^*^ is used to denote a significant difference between groups.

**Figure 4:**
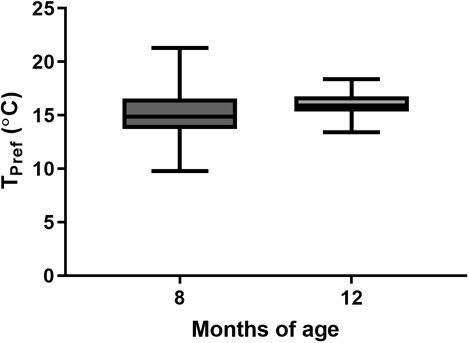
Thermal preference does not change during the juvenile stage of lake whitefish. Boxplots comparing thermal preference (*T*_pref_) of 8- and 12-month-old lake whitefish (LWF) after experiencing 2°C during embryogenesis. Lower and upper box boundaries are the 25th and 75th percentiles of the data, with the line inside the box representing the median, and error lines encompassing the entirety of the spread of data.

As LWF and RWF reside in similar habitats, we sought to assess whether species-specific differences existed in *T*_pref_. LWF reared in a similar condition to RWF (2°C) exhibit an increased preference for warmer waters at 12 months of age (Fig. 3; *t* test, *P* < 0.0001). To investigate the effect of age on *T*_pref_, we compared 8-month-old and 12-month-old LWF incubated at the standard temperature of 2°C. The average *T*_pref_ for 8-month-old LWF was 16.04 ± 1.14°C compared with 15.27 ± 2.67°C for 12-month-old LWF (Fig. 4), which were not statistically different (*t* test, *P* = 0.147).

## Discussion

Our results reveal that elevated temperatures during rearing lead to long-term changes to the *T*_pref_ of RWF, but not LWF. Early-life thermal history has been shown to modulate several plastic traits, including behaviour, social skills and endocrine responses in calves (Dado-Senn *et al.*, 2022), pigs (Johnson *et al.*, 2018) and fish (Jonsson and Jonsson, 2019; Li *et al.*, 2021). While several studies have linked acute or continuous changes in temperature as the aetiology of behavioural and growth changes in fish (Gillooly and Dodson, 2000; Nowicki *et al.*, 2012; Nytrø *et al.*, 2014; Sun and Chen, 2014; Bartolini *et al.*, 2015), studies linking solely early-life incubation temperature to long-term perturbations or functional limitations are sparse (Scott and Johnston, 2012; Jonsson and Jonsson, 2019). Here, we provide evidence that the resulting preference of temperature for fish at the juvenile stage can be programmed during embryogenesis, but this phenotypic plasticity is species specific, and the mechanisms behind these changes are abstruse.

Most teleost fish function as ectotherms, requiring a conserved and coordinated suite of physiological and behavioural responses to navigate changes in ambient temperature (Stevens and Fry, 1970; Haesemeyer, 2020). For example, adults can preferentially swim to more optimal habitats when encountering thermal stress, such as effluent from power plants (Neill and Magnuson, 1974). These behavioural responses are key, allowing animals to maintain their metabolic optimums (Haesemeyer, 2020). At the embryonic stage, fish would be subjected to environmental temperatures without a recourse to navigate to more appropriate conditions. Several studies have described impacts of elevated incubation temperature during embryogenesis, with clear delineations of reductions in survivability and growth, but also in perturbed metabolism (Bozek *et al.*, 1990; Marty *et al.*, 1990; Barrionuevo and Burggren, 1999). In response to direct increases in ambient temperature, there is a positive linkage to metabolism (Fry and Hochachka, 1970). LWF embryos incubated at constant elevated temperatures display increased oxygen consumption (Eme *et al.*, 2015), but it is unknown whether this difference in metabolism persists to the juvenile stage in this species. Other studies have reported increases in post-embryonic metabolism following embryonic incubations in elevated water temperatures, such as in the razorback sucker (*Xyrauchen texanus*; Bozek *et al.*, 1990), zebrafish (*Danio rerio*; Barrionuevo and Burggren, 1999), Arctic charr (*Salvelinus alpinus*; Huuskonen *et al.*, 2003) and Japanese medaka (*Oryzias latipes*; Marty *et al.*, 2010). This suggests that the previously noted increase in metabolism (Eme *et al.*, 2015) may persist to later life stages. This is an important point, as a functional link between basal metabolic rate and thermal preference has been established in the common minnow (*Phoxinus phoxinus*), demonstrating that fish with higher metabolic rates may prefer colder temperatures as juveniles (Killen, 2014). While this could then suggest that RWF reared at 0.5°C have a lowered metabolism, future studies would be required to confirm whether higher metabolism is at the root of lower *T*_pref_ in RWF reared at 2 and 6°C.

Round whitefish appear to be more sensitive to elevations in rearing temperature than LWF. We originally hypothesized that increased temperature during incubation would lead to alterations in *T*_pref_, based upon previous observations that elevated ambient temperature increases mortality in these species (Brooke, 1975; Lee *et al.*, 2016; Lim *et al.*, 2017; Mueller *et al.*, 2017; Eme *et al.*, 2018; Lim *et al.*, 2018; Mitz *et al.*, 2019). At 2°C, RWF experience nearly 30% increase in mortality compared with LWF, with no embryos surviving continuous exposure to 8°C (Lim *et al.*, 2018). RWF appear to exhibit considerable sensitivity to thermal challenges, forming our rationale to reduce the thermal regime RWF were exposed to in this study (6°C) and to include the 0.5°C incubation group. Serendipitously, this reduction revealed that 2 and 6°C appear to be capable of imparting long-term alterations to *T*_pref_ behaviour. This possibly presents an advantage in natural settings to whitefish reared in colder water, as fish experiencing a lower temperature during embryogenesis would then prefer a higher ambient temperature at the juvenile stage. A higher temperature is typically found higher in the water column and may present a greater food supply. Indeed, zooplankton, a major food source for larval and juvenile whitefish (Freeberg *et al.*, 1990), is commonly found in higher abundances at warmer and shallower water (Berger *et al.,* 2006). Taken together, rearing in colder water might provide slight behavioural advantages for RWF, suggesting that ever-increasing ambient temperature driven by anthropogenic practices may be detrimental for this species.

Apart from temperature stressors, RWF appear to be more sensitive to environmental perturbations than LWF. Population declines have been observed in RWF in the New York State (Bouton and Stegemann, 1993; Conley *et al.*, 2021), leading to these fish being labelled vulnerable in this state (Bouton and Stegemann, 1993). Comparing RWF to LWF, the former has historically had a smaller distribution than LWF in North America, with LWF distribution extending farther south beyond the Great Lakes (Ebener *et al.*, 2008). While the specific causes of these declines are unknown, others have speculated this could be explained by the general sensitivity of RWF to abiotic stressors. For example, acid rain has impacted the Adirondack lakes of the New York State, lowering pH and increasing aluminium and mercury, which interfere with reproduction and survival in these fish (Conley *et al.*, 2021). Moreover, exposure to morpholine, a chemical used to prevent corrosion and damage to water pipes and used as an additive in fossil fuels, leads to increased mortality and reduced body size in RWF when compared with LWF at supra-environmental levels (Thome *et al.*, 2016; Lim *et al.*, 2018). In this study, comparisons between LWF and RWF revealed that when raised at a similar temperature, LWF exhibit a higher *T*_pref_ than RWF (Fig. 2). As adults, LWF occupy deeper (18–90 m) limnetic water, with RWF residing in shallow littoral depths (Rawson, 1951; Bailey, 1964; Cucin and Regier, 1965). However, as larvae and juveniles, round and lake whitefish are found feeding along shorelines in shallow water before gradually moving to deeper waters (Faber, 1970; Hogman, 1971). The difference in preferred temperature may support the observation of these species overlapping, but occupying distinct niches and resources (Eberts *et al.*, 2016).

We originally suspected that changes in size and age may play a role in determining *T*_pref_ of whitefish. Previous studies have shown a significant relationship among these variables and preference of temperature in LWF, with *T*_pref_ decreasing as the animal grew/age (Opuszynski, 1974; Edsall, 1999). In this study, we performed assessments to investigate both factors using LWF, performing a regression for *T*_pref_ compared with size (weight and length) and directly comparing the 8- and 12-month-old age class exposed to a similar ambient temperature during embryogenesis (Fig. 3). While we note no correlations of *T*_pref_ with either size or age, we must acknowledge substantial differences of our study design with previous studies investigating this species. Life stage plays a significant role in determining the preference of the animal, as their natural history dictates a transition to deeper waters as the animal ages (Hogman, 1971). In the work by Edsall (1999) and Opuszynski (1974), thermal preference was ascertained by using considerably younger and smaller LWF. This is a key point, as their comparisons were carried out using fish separated by approximately 6–7 months (Opuszynski, 1974; Edsall, 1999), a larger difference than the present study with a 4-month difference in age. Inherently tied to this age difference is a difference in growth, as our study generated a ~10-fold increase in weight from 8 to 12 months of age, and the previous studies describing a more modest increase of ~2- to 3-fold (Opuszynski, 1974; Edsall, 1999). Changes in growth can easily be attributed to holding temperature of post-hatch fish, with our study implementing a common garden temperature of 15°C, compared with the colder holding temperature used previously (8–11°C, Edsall, 1999). This is an important consideration, as despite a substantially larger increase in absolute size in our study, we note no differences in *T*_pref_. This leads to the proposal that developmental age plays a more significant role in determining temperature preferences in LWF. Plankton tow data point to whitefish migrating from warmer coastal waters to cooler and deeper waters at approximately 4 months of age (Loftus, 1982; Ryan and Crawford, 2014), which may suggest that the age classes investigated in this study may have surpassed windows of overt change in *T*_pref_. Another important difference between these studies is the implementation of a vertical testing chamber (Opuszynski, 1974; Edsall, 1999), compared with the horizontal shuttle box used here. In vertical chambers, the coldest temperature is at the bottom of the arena, which may present a behavioural factor that was not considered in our design. In novel situations, fish will exhibit more bottom dwelling type behaviour (Blaser and Rosemberg, 2012), which may drive a larger preference for colder temperature. A comparison between these behavioural paradigms would be prudent for understanding how an animal’s innate response during assessment may influence thermal preferences.

There appears to be no changes in activity levels of LWF and RWF. While the shuttle box is not purposefully built to assess levels of general swimming, movement in this assessment may be considered a gauge of the animal’s exploratory behaviour to seek an optimal environment. In search of a preferred temperature, LWF and RWF of all age classes move to equivalent levels and exert a similar number of chamber transitions across all temperature treatments. Increases in temperature lead to increases in locomotion and anxiety-like behaviours (Biro *et al.*, 2010; Angiulli *et al.*, 2020), with evidence suggesting that the imprinting of temperature in early life can lead to long-term changes in behavioural responses (Li *et al.*, 2021). While we did not explicitly assess general swimming in LWF and RWF, the results generated here suggest that early-life rearing temperature does not effectively alter behaviour during thermal testing.

In conclusion, this study demonstrates a persistent effect of increased embryonic incubation temperature on the thermal preference of juvenile RWF. Benthic water temperatures of 2°C represent a winter of low ice cover but are sufficient to alter preferences in these fish. Given that temperatures are expected to increase (0.1°C/year; Austin and Colman, 2007) and thermal effluents impact coastal water temperature, these results raise concern for fish species that have been considered in decline (Bouton and Stegemann, 1993; Conley *et al.*, 2021) and are rarely seen in abundant amounts ecologically (Mraz, 1964). Coastal embayments provide a thermal refuge during the spring warming (Ryan and Crawford, 2014), and ice-free conditions facilitate a spring bloom of primary productivity, which is important for survival of larval whitefish (Faber, 1970). Round whitefish seeking cooler water temperatures may avoid prime nursery grounds, which would put them at a disadvantage compared with other conspecifics. Cold-adapted freshwater fish are among the taxa most vulnerable to climate change but receive a fraction of the research and conservation efforts of terrestrial species (Pacifici *et al.*, 2015). This study highlights the importance of examining sub-lethal thermal effects and thermal plasticity of cold-adapted species. Future studies seeking to understand the role of metabolism on thermal preference are prudent, as this technique provides a non-invasive assessment of environmental performance that may be used to determine at-risk populations environmentally.

List of Symbols/Abbreviations.

LWF—Lake Whitefish (*Coregonus clupeaformis*).

RWF—Round Whitefish (*Prosopium cylindraceum*).

## Supplementary Material

Web_Material_coad067

## Data Availability

Data and code are available from GitHub: https://github.com/WilsonToxLab/Shuttlebox-Thermal-Preference.
