# Supplementary material for: Effect of elevated embryonic incubation temperature on the temperature preference of juvenile lake (Coregonus clupeaformis) and round whitefish (Prosopium cylindraceum)
Source: Conserv Physiol. 2023 Aug 30;11(1):coad067. doi: 10.1093/conphys/coad067 (PMC10469578; doi:10.1093/conphys/coad067)
Supplement: Web_Material_coad067 [file web_material_coad067.pdf]

Effect of elevated embryonic incubation temperature on the temperature preference of juvenile lake (*Coregonus clupeaformis*) and round whitefish (*Prosopium cylindraceum*)

Adam A. Harman[1], Hannah Mahoney[1], William Andrew Thompson[1], Meghan L.M. Fuzzen[1], Bhuvan Aggarwhal[1], Lisa Laframboise[1], Douglas R. Boreham[2], Richard G. Manzon[3], Christopher M. Somers[3], Joanna Y. Wilson[1]

[1]Department of Biology, McMaster University, 1280 Main St. West, Hamilton, ON L8S 4K1, Canada

[2]Medical Sciences, Northern Ontario School of Medicine, Laurentian University, 935 Ramsey Lake Road, Sudbury, ON P3E 2C6, Canada

[3]Department of Biology, University of Regina, 3737 Wascana Parkway, Regina, Saskatchewan, S4S 0A2, Canada

Corresponding Author: Joanna Y. Wilson (joanna.wilson@mcmaster.ca)

Supplemental Figure 1. Shuttlebox Thermal Preference Set Up. Overhead (A) and side (B) views of the shuttlebox thermal preference chamber (red box in A) and associated set up. Water was maintained in two insulated containers for cold and hot water baths. The cold water bath was kept at temperature by use of an external chiller (labelled in A, but device is out of frame; labelled and visible in B). The chiller was a ¼ HP chiller, magnetic drive centrifugal pump (300W-950W @ 0 – 20°C) operating with 50% ethylene glycol and 50% dechlorinated water set to 4°C. The hot water bath had two (400W) submersed aquarium heaters to maintain temperature at 28°C. Water from the hot and cold buffer tanks circulated through the appropriate water bath through tubing and metal heat exchange coils to control the temperature in the buffer reservoirs. The tubing and buffer tanks were insulated to limit heat/cold exchange with the surrounding air. The water from the buffer tanks was added to each side of the shuttlebox to control the temperature in each side (Δ2°C across sides in this experiment) via the DAQ (data acquisition instrument), which receives information from the temperature probes and controls the pumps to each side. In static mode, the temperatures were 14 and 16°C. In dynamic mode, the movement of the fish determined the temperature change within a set range (see materials and methods for minimum, maximum, hysteresis, and rate). Fish movement was recorded by an overhead camera (B). A screen capture of the overhead video (C) is shown with the fish location identified by a green cross. Fish can freely move between the two circular sides of the arena by a middle rectangular shuttle (demarked by blue and red lines). The software detects and records the movement of fish throughout the arena including the movement of fish across the shuttle (i.e. to change to the other temperature), and the temperature in each side, through time.

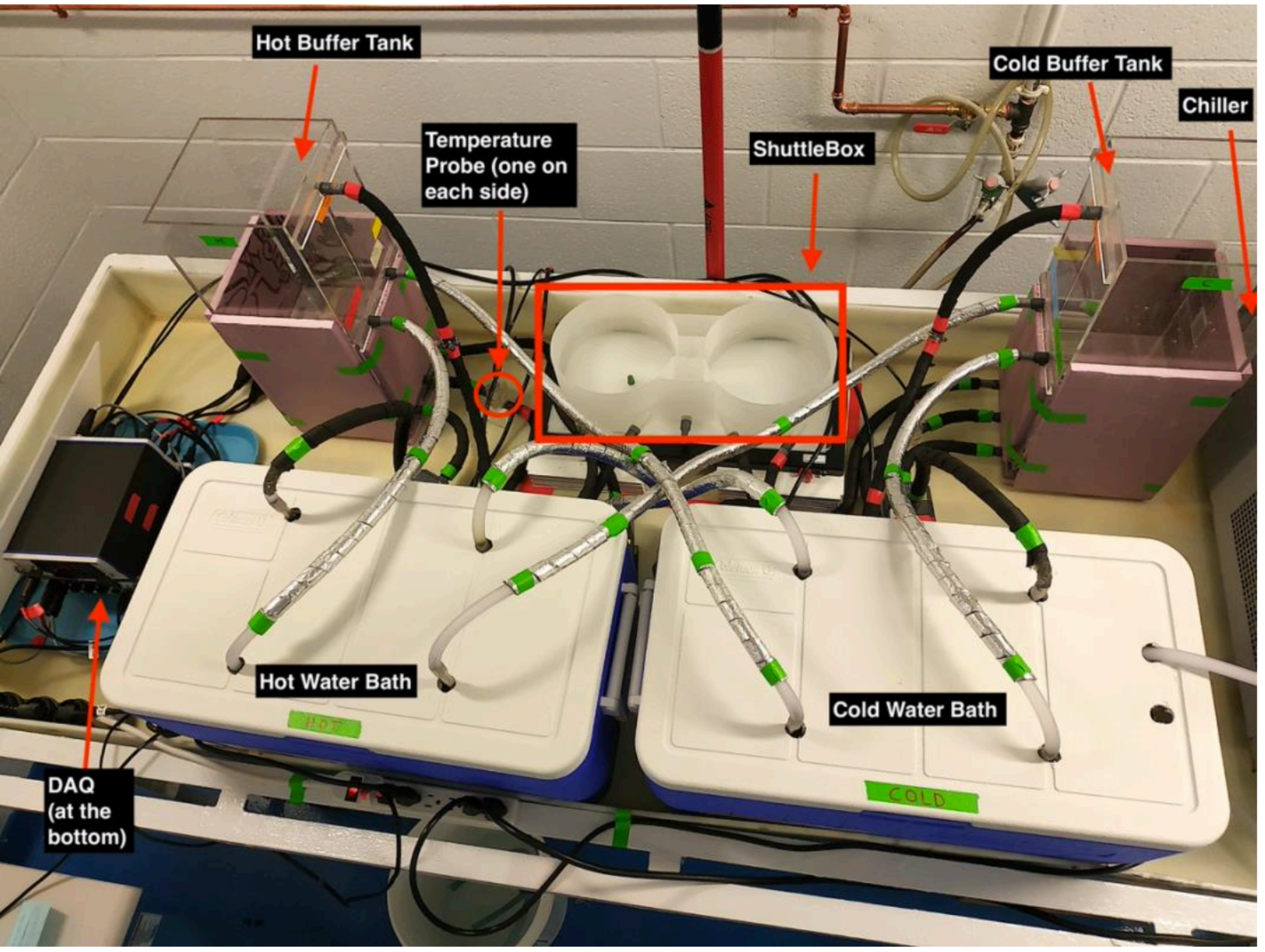
Hot Buffer Tank
Temperature Probe (one on each side)
ShuttleBox
Cold Buffer Tank
Chiller
Hot Water Bath
Cold Water Bath
DAQ (at the bottom)
HOT
COLD

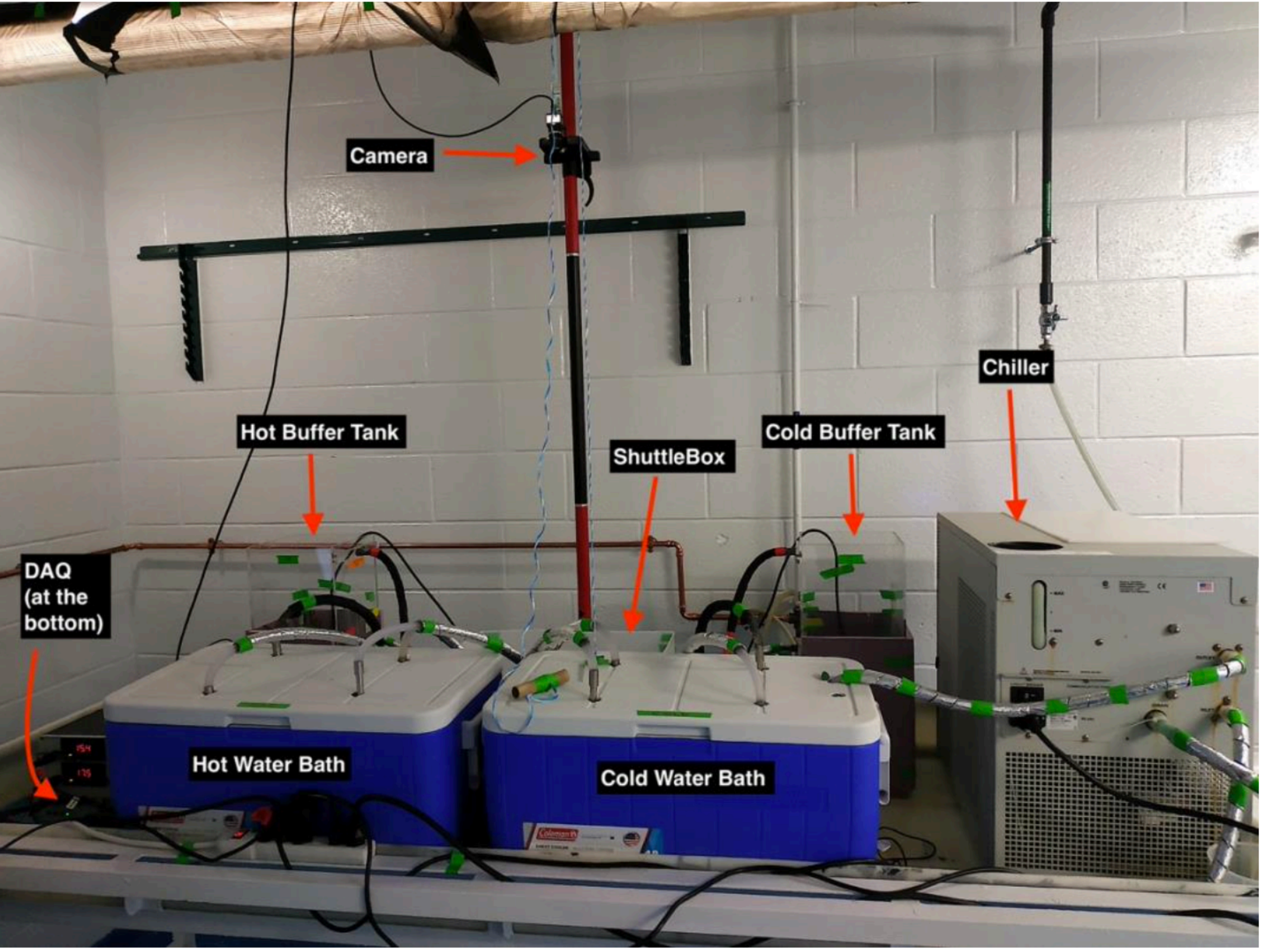

Camera
Chiller
Hot Buffer Tank
ShuttleBox
Cold Buffer Tank
DAQ
(at the
bottom)
Hot Water Bath
Cold Water Bath

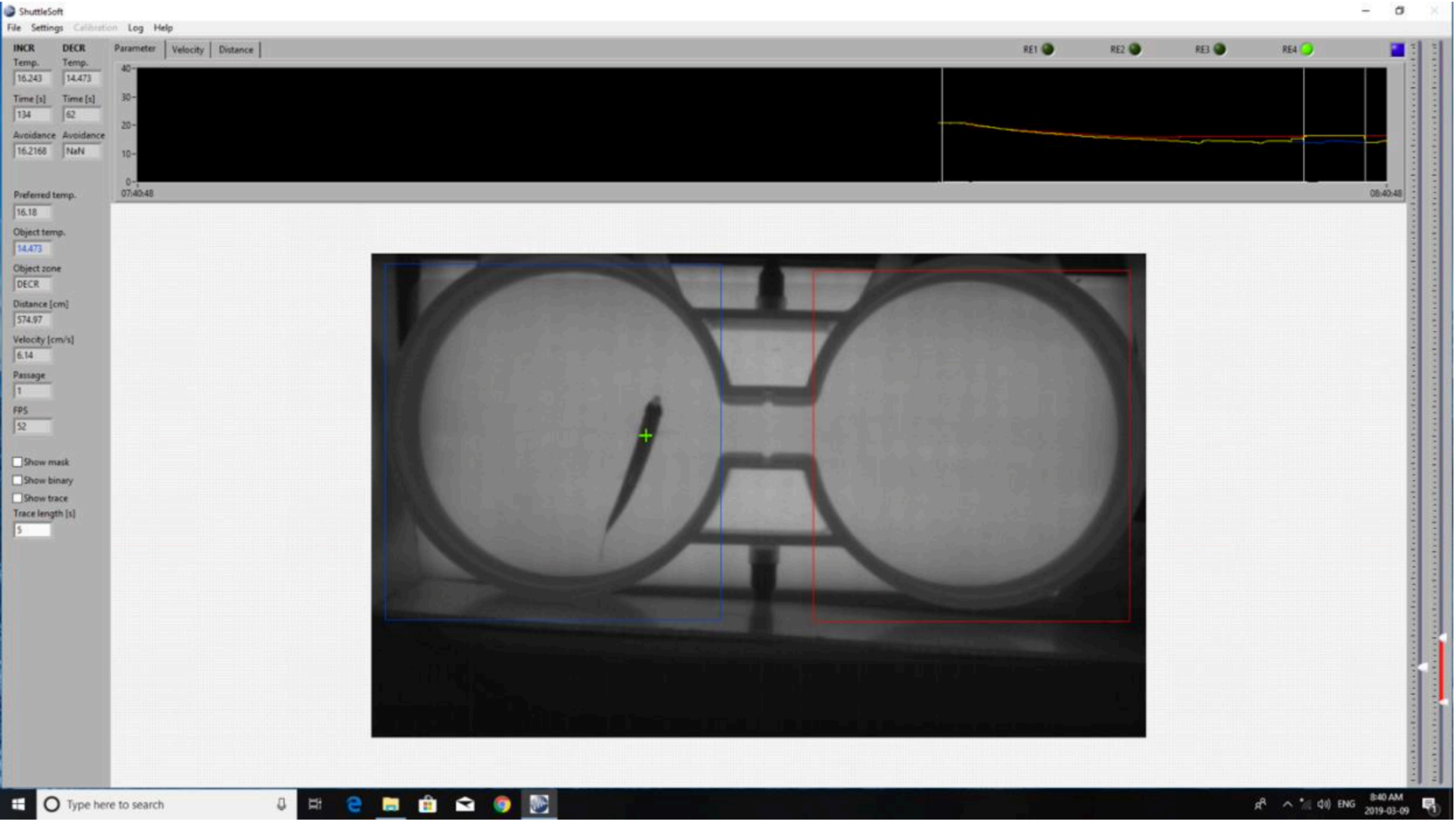
ShuttleSoft
File Settings Calibration Log Help
INCR
DECR
Temp.
16.243
Temp.
14.473
Time [s]
134
Time [s]
62
Avoidance
16.2168
Avoidance
NaN
Preferred temp.
16.18
Object temp.
14.473
Object zone
DECR
Distance [cm]
574.97
Velocity [cm/s]
6.14
Passage
1
FPS
52
Show mask
Show binary
Show trace
Trace length [s]
5
Parameter
Velocity
Distance
RE1
RE2
RE3
RE4
40
30
20
10
0
07:40:48
08:40:48
Type here to search
ENG
8:40 AM
2019-03-09
